# Achromatic super-oscillatory lenses with sub-wavelength focusing

**DOI:** 10.1038/lsa.2017.36

**Published:** 2017-09-08

**Authors:** Guang Hui Yuan, Edward TF Rogers, Nikolay I Zheludev

**Affiliations:** 1 Centre for Disruptive Photonic Technologies, The Photonic Institute, School of Physical and Mathematical Sciences, Nanyang Technological University, Singapore 637371, Singapore; 2 Optoelectronics Research Centre and Centre for Photonic Metamaterials, University of Southampton, Highfield, Southampton SO17 1BJ, UK; 3 Institute for Life Sciences, University of Southampton, Highfield, Southampton SO17 1BJ, UK

**Keywords:** achromatic, super-oscillation, super-resolution

## Abstract

Lenses are crucial to light-enabled technologies. Conventional lenses have been perfected to achieve near-diffraction-limited resolution and minimal chromatic aberrations. However, such lenses are bulky and cannot focus light into a hotspot smaller than a half-wavelength of light. Pupil filters, initially suggested by Toraldo di Francia, can overcome the resolution constraints of conventional lenses but are not intrinsically chromatically corrected. Here we report single-element planar lenses that not only deliver sub-wavelength focusing, thus beating the diffraction limit of conventional refractive lenses, but also focus light of different colors into the same hotspot. Using the principle of super-oscillations, we designed and fabricated a range of binary dielectric and metallic lenses for visible and infrared parts of the spectrum that are manufactured on silicon wafers, silica substrates and optical fiber tips. Such low-cost, compact lenses could be useful in mobile devices, data storage, surveillance, robotics, space applications, imaging, manufacturing with light and spatially resolved nonlinear microscopies.

## Introduction

Chromatic aberration and the resolution limit are two major challenges for high-performance optical imaging. Chromatic aberration (chromatism) is a failure of the lens to focus all colors on the same point. In refractive focusing devices, it results from dispersion of the lens material. In diffractive focusing devices, chromatic aberration results from the accumulated wavelength-dependent phase delay of the electromagnetic waves that form the focus. To reduce chromatic aberrations, complex optical elements such as achromatic doublet, triplet and diffractive-refractive hybrid lenses have been built^1, 2^ with components of opposing dispersion properties^3^. However, such lenses are inevitably bulky, which complicates integration. Several approaches have been proposed to miniaturize beam-shaping and focusing devices including planar Fresnel zone plates and, more recently, metasurface-based plasmonic and dielectric lenses and axicons^4, 5, 6, 7, 8, 9, 10, 11, 12^. However, chromatism remains a key challenge, which has been tackled using dielectric metasurfaces^13, 14^ and wavelength-independent geometric phases^15^ and by exploiting the inherent dispersion in diffractive optics^16^.

The other key parameter of an imaging system is its spatial resolution: the ability to resolve details of the object that is being imaged. It is commonly believed that the resolution of an optical system that images objects from the far-field to the far-field of the lens is limited to half the optical wavelength (the Abbe–Rayleigh diffraction limit) due to the loss of fine detail for the electromagnetic field distribution near the object. Indeed, the Abbe–Rayleigh diffraction limit was viewed as the main obstacle to the development of sub-wavelength-resolution label-free microscopy. Powerful alternative approaches for super-resolution fluorescent bio-imaging have been developed. These include stimulated emission depletion microscopy, which uses two beams, one to excite and the other to deplete luminescence^17^; photo-activated localization microscopy; and stochastic optical reconstruction microscopy, which is based on localizing single luminescent molecules in the imaged object^18, 19^. A common feature of these techniques is the use of fluorescent labels embedded in the object that make them suitable for only a narrow group of applications, predominantly in biology.

Many important technological challenges, from next-generation lithography to data storage and fabrication with light, could be solved if a focusing system existed that could beat the Abbe–Rayleigh diffraction limit and create hotspots much smaller than the wavelength of light. The existence of the resolution limit has been challenged by the promising idea of a far-field to far-field superlens fabricated from a material with a negative index of refraction^20^. Such a superlens could ‘translate’ both the propagating waves and evanescent waves in the immediate proximity of the object into the remote image. Although simpler devices imaging from the near-field to the near-field^21^ and from the near-field to the far-field^22, 23^ have been successfully demonstrated, a negative index superlens that images from the far-field to the far-field has not yet been developed for the optical part of the spectrum and would likely inherit chromatic aberration from the dispersion of the negative index medium.

As originally proposed by Toraldo di Francia, focusing beyond the Abbe–Rayleigh diffraction limit can be achieved by a pupil filtering technique^24, 25, 26, 27^. From the perspective of modern wave theory, this approach exploits the phenomenon of optical super-oscillations^28, 29, 30, 31, 32, 33, 34, 35, 36, 37, 38^, in which a complex band-limited signal can locally oscillate much faster than its highest Fourier components^39, 40, 41^, and thus the accurately tailored interference of waves can form foci smaller than the size allowed by the Abbe–Rayleigh diffraction limit. Mathematically, according to MV Berry, this can be explained by the fact that ‘In the Wigner representations of the local Fourier transform in the ‘phase space’ Wigner function can have both positive and negative values, which causes subtle cancellations in the Fourier integration over all of the function’^42^. In super-oscillatory focusing and imaging devices, fine details of the electromagnetic field near the object are conveyed to the image by the propagating waves themselves, an effect the conventional Abbe–Rayleigh theory deemed impossible since the spatial spectrum of free-space waves is limited by the free-space wavevector.

**Figure 1:**
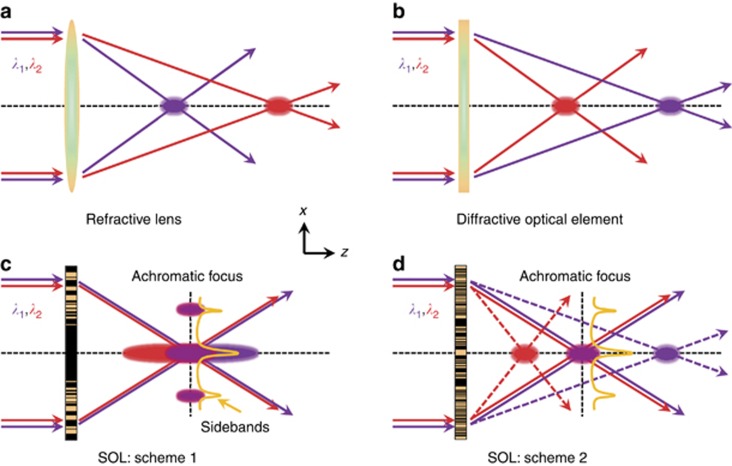
Achromatic SOLs schemes and comparison with conventional refractive lens and diffractive optical element. (**a**) Dispersion of refractive lens (larger focal length for longer wavelength). (**b**) Opposite dispersion of diffractive optical element (larger focal length for shorter wavelength). (**c**) Optical SOL for achromatic focusing: two long depth-of-focus foci overlap to form the achromatic focus. (**d**) SOL focuses two wavelengths *λ*_1_ and *λ*_2_ into multiple discrete hotspots along the axial direction that spatially overlap to achieve bi-chromatic focusing.

This work is devoted to the development of achromatic super-oscillatory sub-wavelength focusing devices. We explore planar masks that convert light waves into super-oscillatory foci by the use of tailored interference of waves diffracted from different areas of the mask (termed a ‘super-oscillatory lens’, SOL). To create super-oscillatory foci, both amplitude and phase masks can be used. Although the outcome of interference is wavelength-dependent, an SOL can be designed to focus different wavelengths into the same spot (Figure 1). This is possible because the SOL can create foci of extremely long depths, extending tens of wavelengths^33, 43, 44^, and so foci of different wavelengths can partially overlap, creating a zone of distances from the SOL where a range of colors can be focused simultaneously. An alternative approach is to use a super-oscillatory mask that generates several discrete foci at different distances from it. For different wavelengths, some of these foci can overlap, and so the SOL will focus two or more wavelengths simultaneously in one spot.

Below, we will describe a range of SOLs, demonstrating the wide variety of lenses that can be designed and the flexibility possible in designing lenses for particular applications. We have made both amplitude- and phase-modulated achromatic SOLs for visible and infrared radiation and an apochromatic red/green/blue SOL for the visible part of the spectrum. Here and below, we adopt the well-established terminology^1, 2, 3^ that a lens is called achromatic if it brings two wavelengths into focus in the same plane and apochromatic if it focuses three wavelengths simultaneously.

## Materials and methods

### Achromatic SOL design procedure

For the design of achromatic SOLs, we used the multi-objective particle swarm optimization (PSO) algorithm^45^, which optimizes a problem regarding a given merit function using a population of ‘particles’ in the *N*-dimensional search space. For a ring mask, the radial direction is divided into *N* equally spaced zones. Each zone has either a unit (‘1’) or zero (‘0’) value, which corresponds to transparent and opaque for the amplitude mask, and two phase levels for the phase mask. The PSO algorithm searches for the best arrangement of these binary values. In contrast to our previous works^30, 33, 36^, here, the target function to describe the electric field intensity profiles near the focus for individual wavelength *λ*_*p*_ is defined as


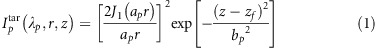


where *p* is the numbering of wavelengths, *J*_1_(*x*) is the first-order Bessel function of the first kind, *z*_*f*_ is the desired achromatic working distance, 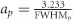, 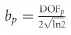, FWHM_*p*_ is the full-width half-maximum (FWHM) of the transverse hotspot size, and DOF_*p*_ is the depth of focus at *λ*_*p*_. The merit function to achieve the achromatic SOL design is given as


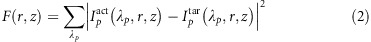


where 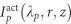 is the normalized actual intensity distribution at *λ*_*p*_ and is calculated by the angular spectrum method for a given mask design. The optimal SOL mask is achieved after sufficient iterations to allow *F*(*r*,*z*) to reach its minimum value.

### SOL sample preparation and fabrication

All SOLs were fabricated by focused ion beam milling (Helios 650, Hillsboro, OR, USA). For the binary amplitude mask, a 100-nm-thick gold film was deposited on a silica glass substrate using a thermal evaporator (Oerlikon Univex 250, Cologne, Germany) with a deposition rate of 0.2 Å s^−1^. Prior to deposition of the gold, a 5-nm-thick chromium adhesion layer was deposited on the substrate. For the fiberized SOL, the fabrication process remains essentially the same, while the photonic crystal fibers (LMA35, NKT Photonics, Birkerød, Denmark) are cleaved with a large-diameter fiber cleaver (VYTRAN LDC-400, Thorlabs Inc., Newton, NJ, USA). A careful alignment between the centers of the SOL and fiber core must be achieved for correct performance of the SOL. For the dielectric SOL, FIB writing is conducted directly onto the silicon wafer (University Wafer Inc., DSP, 100, p-type).

## Results and discussion

### Near-IR achromatic fiberized amplitude mask SOL

A sub-wavelength focusing, near-IR lens on a fiber tip would be valuable in many applications, most notably imaging through silicon wafers and substrates inside silicon chips, diagnostics of optoelectronic devices, high-resolution two-photon-polymerization nano-fabrication, non-destructive imaging of *in vitro* biomedical samples, micro-spectrometry of molecular vibrational modes, and an interconnect element of silicon photonic devices. We chose the tip of a single-mode large-mode-area photonic crystal fiber (PCF, NKT Photonics) as the platform for our achromatic near-infrared SOL. The advantage of such fibers is that they provide a stable high-quality mode, even at high output power, and single-mode operation with almost constant mode diameter for broadband wavelengths, which is useful—though not essential—when designing achromatic devices.

**Figure 2:**
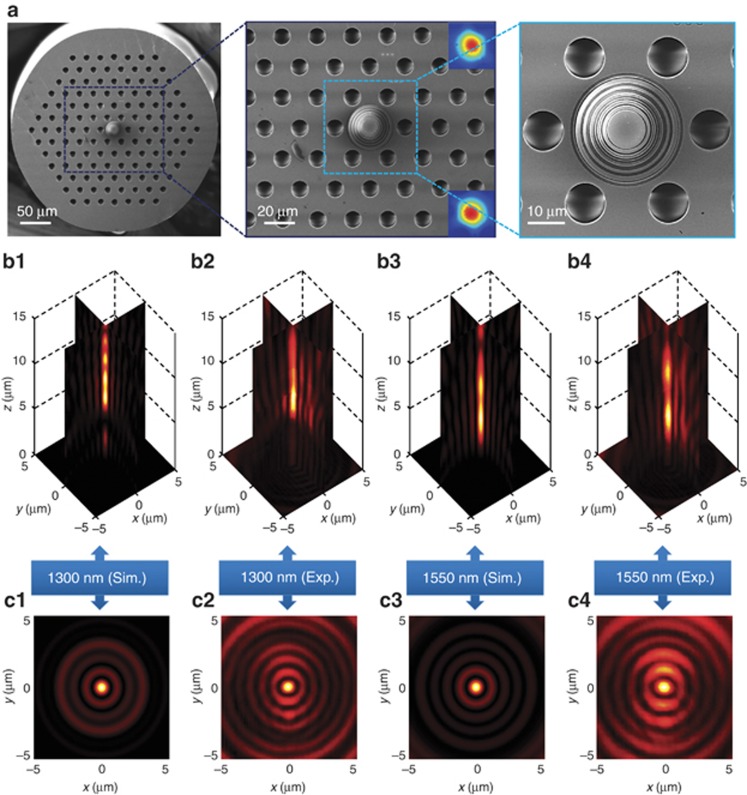
Near-IR achromatic fiberized amplitude-mask SOL. (**a**) SEM image and zoomed-in views of the SOL manufactured on the core of a single-mode large-mode-area photonic crystal fiber in which a 100-nm-thick gold film was deposited on the fiber end after cleaving. Insets in the middle figure show the fiber modes at the wavelengths of *λ*_IR1_=1.3 μm (top) and *λ*_IR2_=1.55 μm (bottom). (**b**) Experimental and simulated field patterns in two typical cross-sections, where the achromatic super-oscillatory hotspots are expected at *z*_*f*_=8 μm. (**c**) Corresponding intensity profiles in the achromatic transverse focal plane. Exp., experiment; Sim., simulation.

The SOL is a concentric ring nanostructure located in the middle of the PCF cross-section (see SEM micrograph in Figure 2a). It is optimized to be achromatic at two telecommunication wavelengths *λ*_IR1_=1.3 μm and *λ*_IR2_=1.55 μm. The design and optimization procedures, and the fabrication process, are described in the Materials and Methods section (see Supplementary Information Section 1 for the design parameters of all SOLs used in this paper). The theoretical focusing performance for the chosen wavelengths—calculated by the angular spectrum method^30^—is shown in Figure 2b1 and 2b3. Calculations reveal that at *λ*_IR1_=1.3 μm, the SOL produces an ‘optical needle’, a long focal spot extending from an axial distance of 6.1–11.9 μm, while at *λ*_IR2_=1.55 μm, the ‘optical needle’ extends from 3.1 to 9.7 μm from the lens. Here, achromatic performance can be expected from 6.1 to 9.7 μm, centered at a working distance of ~8 μm.

To experimentally characterize the lens, we first measured the mode field diameter of the fiber and found it to be 26 μm for both wavelengths of interest, *λ*_IR1_=1.3 μm and *λ*_IR2_=1.55 μm, as shown in the inset of Figure 2a. A conventional glass lens with a diameter of 26 μm and focal distance of 8 μm would have a numerical aperture (NA) of 0.85. Since the super-oscillatory field structure of the lens is formed by free-space waves, it can be imaged with a lens of similar or higher numerical aperture. The lens was characterized with a tunable supercontinuum laser source coupled into the fiber and a high-resolution InGaAs camera equipped with an objective of NA=0.95 (see Supplementary Information Section 2 for the experimental details).The intensity pattern after the SOL was recorded slice by slice with an axial step of 200 nm (Figure 2b2 and 2b4). The optical needles are located between 5.4 and 10.8 μm for *λ*_IR1_, and between 3.4 and 10.4 μm for *λ*_IR2_, showing reasonable agreement with simulations. The fractions of energy focused into the sub-wavelength hotspots at the target plane at 8 μm were found to be 2.9% and 2.1% for *λ*_IR1_ and *λ*_IR2_, respectively.

The cross-sections of the hotspots at 8 μm from the lens are presented in Figure 2c. At *λ*_IR1_=1.3 μm, the FWHM in the simulation was 0.47 × *λ*_IR1_ compared to the experimental observation of (0.51±0.02) × *λ*_IR1_. At *λ*_IR2_=1.55 μm, the FWHM of the predicted hotspot was 0.44 × *λ*_IR2_ compared to the experimental observation of (0.48±0.02) × *λ*_IR2_. A normal glass lens of equivalent size would not be able to deliver such sharp focus: its focal spot would be at least the Abbe–Rayleigh diffraction limit of 0.59*λ*.

We used two approaches to prove that the generated fields are actually super-oscillatory: one confirms that the spatial spectra of the hotspots are beyond the available band-limited spectrum of the overall field, and the other shows that the local wavevectors are locally much larger than the highest wavevector of the light (see Supplementary Information Section 3 for the details).

### Near-IR achromatic silicon phase-mask SOL

**Figure 3:**
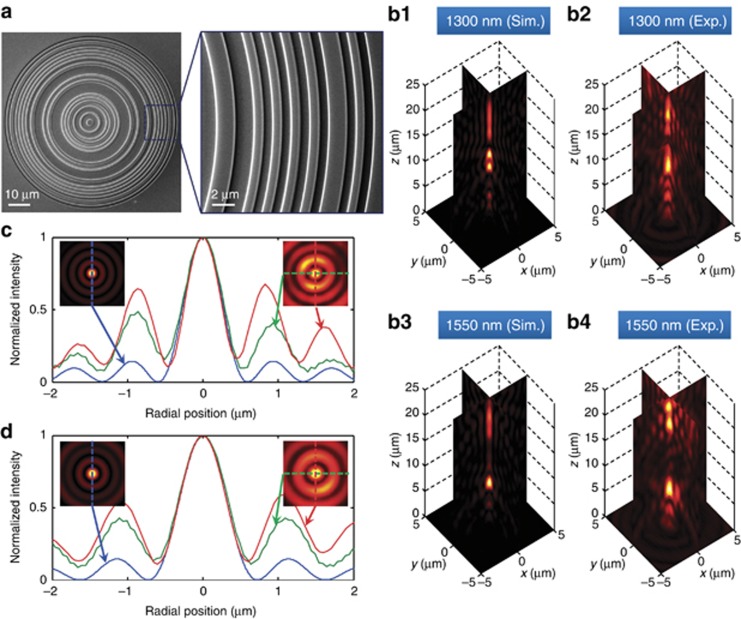
Near-IR achromatic silicon phase mask SOL. (**a**) SEM micrograph of achromatic SOL milled on a silicon wafer, designed for achromatic focusing for wavelengths of *λ*_IR1_=1.3 μm and *λ*_IR2_=1.55 μm with working distance of *z*_*f*_=20 μm. The right zoomed-in view illustrates the fabrication quality. (**b**) Experimental and simulated achromatic focusing behavior in the planes parallel (*xz*) and perpendicular (*yz*) to the incident polarization. (**c**, **d**) Comparison of the hotspot size between simulations and experimental results for *λ*_IR1_ (**c**) and for *λ*_IR2_ (**d**). Insets are intensity profiles at *z*_*f*_=20 μm. Blue curves are data from an angular spectrum simulation. Red and green curves show the line-scan profiles that are perpendicular and parallel, respectively, to the incident polarization (*x*). Exp., experiment; Sim., simulation.

Similar achromatic focusing can be achieved with a dielectric phase mask. Low-loss dielectric phase masks can simultaneously improve the throughput efficiency of the lens and markedly enhance optical breakdown thresholds of the device for both continuous wave and pulsed illumination. We chose to work with silicon because it is compatible with the fabrication process of well-established technologies. Here, we used focused ion beam milling, although high-resolution optical and electron beam lithography would give similar or even better results. By milling different depths into a silicon wafer, we created a lossless dielectric achromatic SOL for the same telecommunication wavelengths of *λ*_IR1_=1.3 μm and *λ*_IR2_=1.55 μm. The refractive indices at *λ*_IR1_ and *λ*_IR2_ were measured to be 3.509 and 3.481, respectively (see Supplementary Information Section 4 for the *n*–*k* curves), and the material’s dispersion was considered during the mask optimization. At a milling depth of 312 nm, a step in the silicon layer creates a phase retardation of 1.2*π* at *λ*_IR1_=1.3 μm and *π* at *λ*_IR2_=1.55 μm. The SEM micrograph of an SOL is shown in Figure 3a.

The computed and experimentally measured focusing performance of the lens is presented in Figure 3b. The super-oscillatory hotspots can be observed near an axial distance of 20 μm, where at *λ*_IR1_, the theoretical depth of focus is 6.7 μm, compared with the experimental value of 5.2 μm. At *λ*_IR2_, we obtained 5.4 and 5.8 μm for the computed and experimentally observed depths of focus, respectively. At 20 μm from the lens, the hotspot size at *λ*_IR1_=1.3 μm was 0.427 × *λ*_IR1_ (simulation, blue curve) and (0.42±0.04) × *λ*_IR1_ (experiment); see Figure 3c. Similarly, for *λ*_IR2_, the FWHM of the hotspot was 0.435 × *λ*_IR2_ (simulation) and (0.44±0.02) × *λ*_IR2_ (experiment); see Figure 3d. The fractions of energy focused into the sub-wavelength hotspots at the target plane at 20 μm were found to be 1.26% and 2.1% for *λ*_IR1_ and *λ*_IR2_, respectively. For comparison, the diffraction limit is 0.56 × *λ*.

### Visible achromatic amplitude mask SOL

Interference-based super-oscillatory focusing is scalable to any wavelength. An SOL working at a shorter wavelength places greater demand on the finesse and accuracy of fabrication, but we show here that it is still possible to manufacture visible-wavelength achromatic SOLs by focused ion beam milling. As examples, we have chosen two wavelengths of *λ*_1_=690 nm and *λ*_2_=870 nm, one in the visible band and the other in the infrared band. These or similar wavelengths are often used in nonlinear optical imaging techniques and spectroscopic pump-probe experiments, particularly with the widespread Ti:sapphire laser sources.

**Figure 4:**
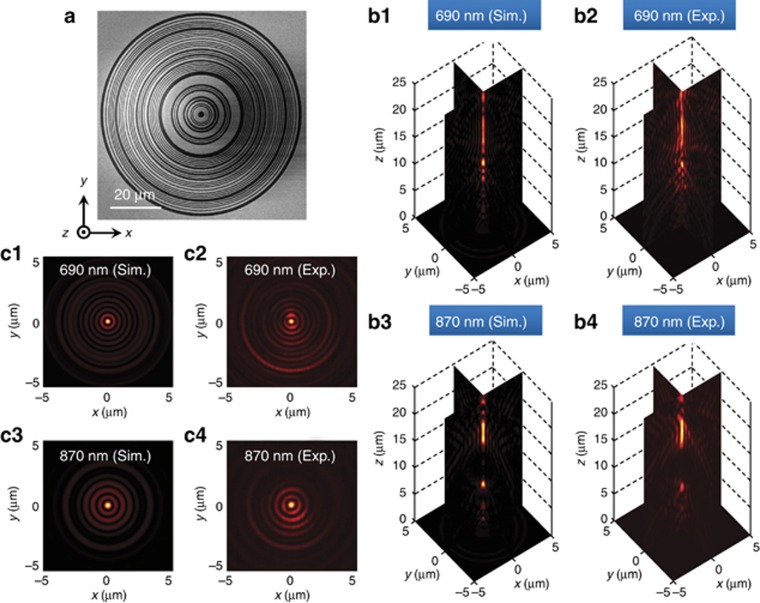
Visible achromatic amplitude mask SOL. (**a**) SEM micrograph of the fabricated mask with diameter of 80 μm and smallest ring width of 400 nm on a 100-nm-thick gold film. (**b**) Experimental and simulated diffraction patterns in the *xz* and *yz* cross-sections, where the achromatic super-oscillatory hotspots are generated at *z*_*f*_=18 μm for *λ*_1_=690 nm and *λ*_2_=870 nm. (**c**) Corresponding field patterns in the achromatic transverse focal plane. The incident beam is *x*-polarized. Exp., experiment; Sim., simulation.

The mask is shown in Figure 4a. Simulated field patterns in the central cross-sections along (*xz* plane) and perpendicular to the incident polarization (*yz* plane) are shown in Figure 4b. The incident beam is an *x*-polarized plane wave. Achromatic performance is centered at *z*_*f*_=18 μm, where an elongated hotspot at *λ*_1_=690 nm (15–20.1 μm) overlaps with a hotspot at *λ*_2_=870 nm (16–19.9 μm) for *λ*_2_. The FWHMs of the hotspots are 280 nm (0.405 × *λ*_1_) and 367 nm (0.422 × *λ*_2_) for *λ*_1_ and *λ*_2_, respectively, and are sub-diffraction-limited. For a conventional lens of this size, the diffraction-limited hotspot (0.548 × *λ*) will be 378 nm at *λ*_1_=690 nm and 477 nm at *λ*_2_=870 nm.

The experimentally measured field structures are presented in Figure 4b2 and 4b4. Hotspots with comparable depths of focus are generated at *z*_*f*_=18 μm, which is in agreement with the theoretical predictions. At *λ*_1_=690 nm, the observed dimensions of the hotspots were 0.42 × *λ*_1_ for the direction along the incident polarization and 0.46 × *λ*_1_ for the perpendicular direction. For *λ*_2_=870 nm, the spot sizes were 0.43 × *λ*_2_ and 0.44 × *λ*_2_ in the two directions, respectively; see Figure 4c2 and 4c4. The fractions of energy focused into the sub-wavelength hotspots at the target plane at 18 μm were found to be 0.47% and 1% for *λ*_1_ and *λ*_2_, respectively.

Here we note that the conventional binary Fresnel zone plate is highly dispersive, and its focal distance will depend on the wavelength, progressively shifting away from the lens for longer wavelengths. Thus, the Fresnel zone plate cannot offer an achromatic solution (see Supplementary Information Section 5).

### RGB ‘white’-light apochromatic amplitude mask SOL

**Figure 5:**
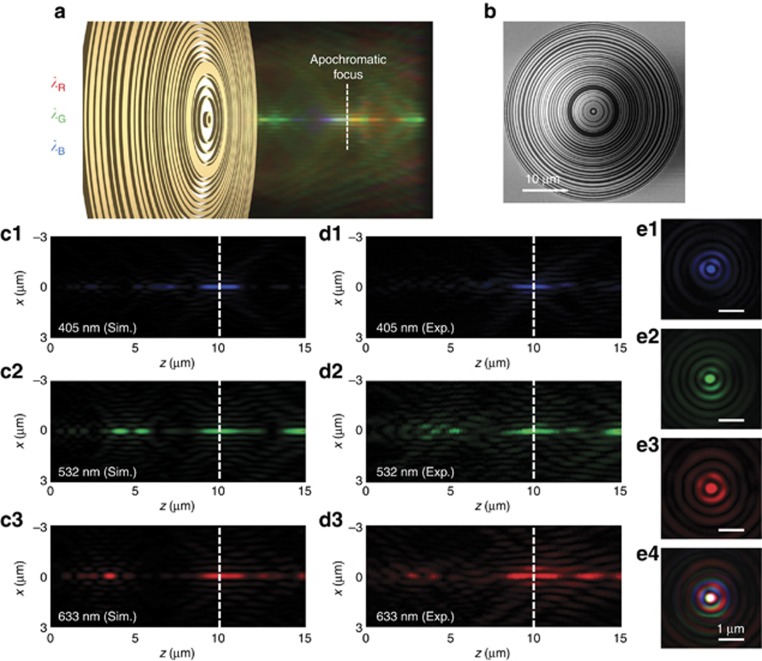
RGB ‘white’-light apochromatic amplitude mask SOL. (**a**) Apochromatic SOL focuses simultaneously at three different wavelengths, red (*λ*_R_=633 nm), green (*λ*_G_=532 nm) and blue (*λ*_B_=405 nm), that can form a ‘white’ super-oscillatory hotspot. (**b**) SEM micrograph of the fabricated mask with a diameter of 40 μm and a working distance of 10 μm. (**c**, **d**) Simulated (**c**) and experimental (**d**) diffraction patterns in the *xz* cross-section. The vertical dashed white lines indicate the focal plane. (**e**) Experimentally registered intensity patterns in the transverse focal plane: (e1) for *λ*_B_, (e2) for *λ*_G_, (e3) for *λ*_R_ and (e4) for RGB wavelengths by simultaneously switching on the three channels. Images are captured by a color CCD camera. Exp., experiment; Sim., simulation.

We found that designing a single-element lens that focuses light across the entire visible range into a single hotspot is difficult (see the previous SOL performance for the intermediate wavelengths between *λ*_1_ and *λ*_2_ in Supplementary Information Section 6). However, we have been able to design an SOL that simultaneously focuses the three primary colors of the additive model that together can produce perceived white light. We have developed an apochromatic SOL for red (*λ*_R_=633 nm), green (*λ*_G_=532 nm) and blue (*λ*_B_=405 nm) light, as illustrated in Figure 5a. It is well known from computer display and digital imaging technology that any perceived color can be generated by combining these three colors.

Figure 5b shows an SEM micrograph of the mask. We targeted a working distance of 10 μm and an FWHM of 0.4 × *λ*, which is 28% less than the diffraction limit of 0.56 × *λ*. The simulated intensity profiles in the longitudinal cross-section are given in Figure 5c. The corresponding experimental data are shown in Figure 5d. The field structures in the focal plane for R, G and B wavelengths are displayed in Figure 5e1-5e3. The simulated hotspot sizes were 0.438 × *λ*_R_ at *λ*_R_=633 nm, 0.422 × *λ*_G_ at *λ*_G_=532 nm and 0.523 × *λ*_B_ at *λ*_B_=405 nm. Experimentally we obtained hotspots of (0.457±0.01) × *λ*_R_, (0.445±0.04) × *λ*_G_ and (0.54±0.03) × *λ*_B_, respectively. The fractions of energy focused into the sub-wavelength hotspots at the target plane were found to be 1.35%, 1.18% and 1.54% for *λ*_R_, *λ*_G_, and *λ*_B_, respectively.

As expected, we could acquire a ‘white’ super-oscillatory hotspot by focusing supercontinuum fiber laser radiation that was spectrally conditioned at the selected RGB wavelengths by a programmable acousto-optic tunable filter; see Figure 5e4. Notably, superachromatic SOLs that work for more than three wavelengths are also possible and can be designed in a similar way. We give an example of an SOL working for four wavelengths in Supplementary Information Section 7.

## Conclusions

We experimentally demonstrated that chromatic aberration and the optical diffraction limit can be simultaneously overcome using an SOL consisting of a single planar optical element. The single-element SOL creates sub-diffraction hotspots by delicate constructive interference of propagating optical waves with different wavevectors and therefore allows foci at different wavelengths to spatially overlap in an achromatic manner.

Sub-wavelength focusing of SOLs comes at a price. Only a small fraction of light incident on the lens is actually focused in the central hotspot, while the remaining fraction is mainly distributed in broad ‘halo’ rings around the hotspot. For some applications, the effect of a halo can be eliminated. For instance, in imaging, light scattered by a halo can be suppressed using a confocal technique^30^, while in heat-assisted magnetic recording applications, it can be illuminated by using additional thin apertures^46^. In some nonlinear optical applications, such as coherent anti-Stokes Raman spectroscopy^47^, suppression of the halo comes naturally through nonlinear interactions at different frequencies. Although we did not consider the energy concentration problem during our SOL design process, it would be possible to incorporate it into the same framework^48^. Increasing the dimension number *N* would give more degrees of freedom for maximization of energy concentration, especially for multiple wavelengths. For phase-type SOLs, increasing the number of phase levels would be helpful to reduce the sidebands and increase the focusing efficiency of super-oscillatory hotspots.

In the focusing devices described here, 1%–3% of incident energy is focused in the central hotspot, while the intensity level could be a factor of 3 higher than the intensity level of the incident wave onto the lens, as for instance, in the fiberized lens described above. The low level of throughput efficiency may be prohibitive for some demanding applications but may be tolerable for others. For instance, a low-loss dielectric SOL, that is, 40 μm in diameter will be able to sustain incident continuous wave laser radiation of a few watts, corresponding to focal intensities of a few hundred kW cm^−2^. When used with pulsed lasers, the lens will be able to sustain a few hundred mJ cm^−2^ of incident fluence with femtosecond and picosecond pulses and up to a few J cm^−2^ with nanosecond pulses^49, 50^. It will deliver a similar level of energy density in the hotspot. Such levels of optical excitations will be overwhelmingly sufficient for raster imaging, lithography and nanofabrication with light.

We fabricated metallic and dielectric binary amplitude and phase masks that are wavelength scalable and can work for different spectral bands. In the infrared band, we implemented achromatic SOLs at the tip of large-mode area single-mode photonic crystal fibers and on silicon wafers. For the visible band, we developed an achromatic SOL for two well-separated wavelengths and an apochromatic SOL for RGB wavelengths suitable for creating a sub-diffraction ‘white’ hotspot.

We anticipate that achromatic sub-diffraction photonic focusing devices could serve as super-resolved focusing and imaging tools for a broad range applications in nonlinear optics, photography, retinal diagnostics^51^, low-cost fiberized microscopy, non-invasive and label-free biological imaging, pump-probe experiments for ultrafast dynamics, excitation and collection of photoluminescence, coherent anti-Stokes Raman scattering for super-resolution bio-imaging, nonlinear imaging and nanofabrication using two/three-photon generation.

## Author contributions

NIZ and GHY conceived the idea of achromatic sub-diffraction focusing using super-oscillations. GHY and ETFR carried out the super-oscillatory lens design and angular spectrum simulations. GHY performed the fabrication, measurements and data analysis. All authors co-wrote the paper, discussed the results and cross-edited the manuscript extensively. NIZ supervised and coordinated all the works.
