# Supplementary material for: Achromatic super-oscillatory lenses with sub-wavelength focusing
Source: Light Sci Appl. 2017 Sep 8;6(9):e17036–. doi: 10.1038/lsa.2017.36 (PMC6062332; doi:10.1038/lsa.2017.36)
Supplement: Supplementary Information [file lsa201736x1.docx]

Supplementary Information for

**Achromatic super-oscillatory lenses with sub-wavelength focusing**

**Guang Hui Yuan^1#^,** **Edward T. F. Rogers^2,3^, and Nikolay I. Zheludev^1,^****^2*^**

*^1^Centre for Disruptive Photonic Technologies,* *The Photonic Institute, SPMS, Nanyang Technological University, Singapore 637371, Singapore*

*^2^Optoelectronics Research Centre and Centre for Photonic Metamaterials,* *University of Southampton, Highfield, Southampton, SO17 1BJ, UK*

*^3^Institute for Life Sciences, University of Southampton, Highfield, Southampton, SO17 1BJ, UK*

^#^ghyuan@ntu.edu.sg and [**^*^**nzheludev@ntu.edu.sg](mailto:*nzheludev@ntu.edu.sg)

**Contents:**

1. Design parameters of achromatic SOLs
2. Experimental setup for SOL characterization
3. Verification of super-oscillatory field
4. Optical refractive index of silicon wafer
5. Comparison between achromatic SOL and binary Fresnel zone plates
6. Evolution of diffraction patterns at intermediate wavelengths
7. Superachromatic SOL design for four wavelengths
8. **Design parameters of achromatic SOLs**

All design parameters of the achromatic SOLs discussed in this paper are shown in Table S1, including working wavelengths, materials and substrates, working distance, size and dimensions.

| **Table S1.** Design parameters of the achromatic SOLs | | | | | | | |
| --- | --- | --- | --- | --- | --- | --- | --- |
| Sample # | Wavelengths (nm) | Material and substrates | Working distance (𝜇m) | SOL size | Dimension (*N*) | Amp/Phase modulation | Binary values (from centre to outward) * |
| 1. Fiberized SOL | 1300/1550 | 100 nm Au on fiber tip | 8 | Dia. 30 𝜇m | 75 | Amplitude | 000000000000000000000000001000001100100110111010011111000011110000001101111 |
| 2. Dielectric SOL | 1300/1550 | Silicon wafer | 20 | Dia. 80 𝜇m | 50 | Phase | 11000100010110101000000110010000011001010101011010 |
| 3. Visible SOL | 690/870 | 100 nm Au on silica glass | 18 | Dia. 80 𝜇m | 100 | Amplitude | 1110000010100110110000011010110000000001111010101010110010110101100101010010010101011101010100100111 |
| 4. RGB SOL | 405/532/633 | 100 nm Au on silica glass | 10 | Dia. 40 𝜇m | 100 | Amplitude | 0011000000010000100001011111101001001001101101101100101001011010011001101100111001100100110110110100 |
| Remark | *The binary values 0 and 1 correspond to zero and unit transmittance for amplitude masks, and two-level phase for phase masks. In the dielectric SOL, the binary phase is 0/1.2𝜋 for 1300 nm and 0/𝜋 for 1550 nm. Both designs take the material dispersion into account during the mask optimization procedure. | | | | | | |

Fig. S1 gives an example of the fiber sample before and after SOL integration, where the hexagonally arranged air holes with a central defect confine the optical mode and a well-aligned and concentrically symmetric multi-ring structure can be clearly seen in the core region.


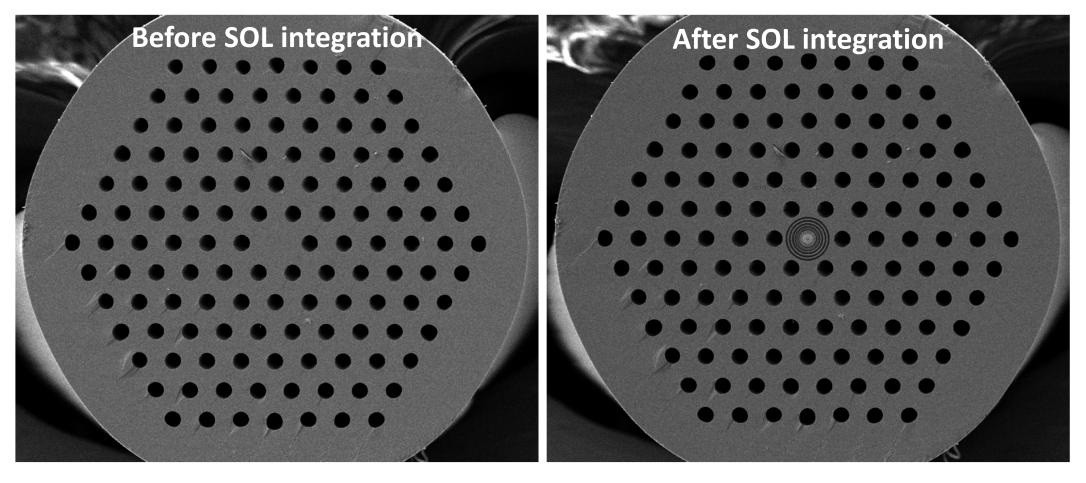


**Figure S1.** Large-mode-area single-mode photonic crystal fiber before (left) and after (right) SOL integration. The fiber end surface is coated with a 100-nm-thick gold film which serves as an amplitude mask.

1. **Experimental setup for SOL characterization**

The experimental setup is sketched in Fig. S2. All the experiments were conducted using a super-continuum laser source (Fianium WL-SC-400-8, UK) with spectral range from 400 nm to 2500 nm. The individual wavelength channel can be selected and turned on/off using compatible acousto-optic tunable filters (AOTF) and with the relative power in each channel controllable by software. After collimation by a fiber coupler (FC1), the beam is directed into a customized dual-mode microscope (Nikon Eclipse Ti-E/LV) using a pair of mirrors (M1, M2) and illuminates the SOLs after reflection by a dichroic mirror (DM) orientated at 45°. Since the super-oscillatory fields are formed by delicate interference of propagating waves, they can be mapped into the far-ﬁeld and directly imaged by a conventional optical imaging system. We used a high-magnification, high-numerical aperture objective (Nikon CFI LU Plan APO EPI 150X, NA=0.95) to collect the diffracted fields which are additionally magnified by a 4X magnification changer (Nikon C-Mount TV Adapter VM 4X) and subsequently imaged by a suitable high-resolution camera (Andor Neo sCMOS camera for 690 nm/870 nm; Thorlabs USB2.0 color CMOS camera for RGB wavelengths; Photonic Science SWIR InGaAs VGA camera for IR wavelengths). The transverse cross-section distributions at specific propagation distances are obtained by *z*-scanning of the SOL mounted on a 3-axis piezo stage (PI P545). The longitudinal cross-section distributions are captured simultaneously from real-time data processing in Labview.

When measuring the fiberized SOL, we simply mount the fiber onto the same sample stage and use similar characterization procedures. The second fiber coupler (FC2) is used to couple the laser beam into a photonic crystal fiber (PCF, ~one-meter) on the other end of which is integrated with the SOL.


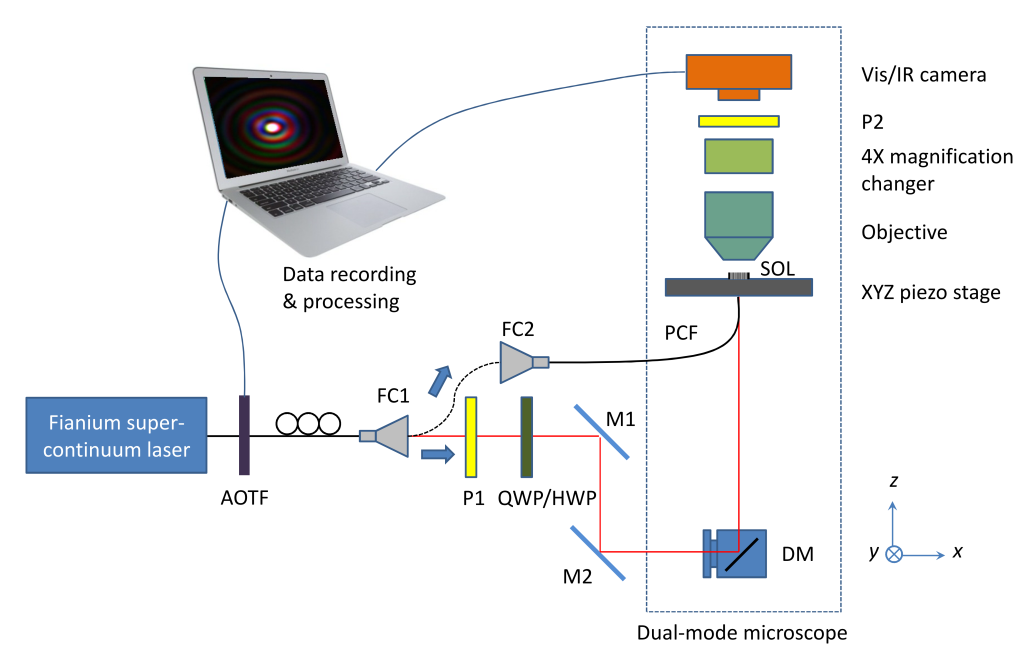


**Figure S2.** Experimental setup for SOL characterization. AOTF: acousto-optic tunable filter; P1, P2: polarizer; M1, M2: optical mirrors; FC1, FC2: fiber couplers; PCF: photonic crystal fiber; DM, dichroic mirror.

1. **Verification of super-oscillatory field**

We use two approaches to confirm the generated optical fields at focus are indeed super-oscillatory for both achromatic wavelengths, here using fiberized SOL as an example.

Method (i): For the overall field in the focal plane, its spatial spectrum is calculated using the two-dimensional Fourier transform

$\tilde{E}\left( k_{x},k_{y} \right)=\frac{1}{2\pi}\iint E\left( x,y \right)e^{-i\left( k_{x}x+k_{y}y \right)}dxdy$ (1)

The results are given in Fig. S3a and Fig. S3c for 𝜆_IR1_=1300 nm and 𝜆_IR2_=1550 nm respectively. It is clearly seen that the spatial spectrum $\left( k_{x},k_{y} \right)$ is band-limited to $\sqrt{k_{x}^{2}+k_{y}^{2}}\leq k_{0}$, where $k_{0}=\frac{2\pi}{\lambda}$ is the free space wavevector, the band-limit is shown by the black dashed circles. For comparison, the spatial spectrum of the hotspot - focal field within the first intensity minimum as shown in Fig. 2c1 and 2c3 in the main text- can be beyond the available spectrum of the overall field, signified by the presence of Fourier components with high values of wavevectors $\sqrt{k_{x}^{2}+k_{y}^{2}}>k_{0}$. This is the origin of the super-resolution of super-oscillatory focusing that an optical field, even in the far-field, can have fine sub-wavelength features in a local area.

Method (ii): We calculated the phase profiles of the electric field at focus from which the local wavevector *k*_local_ is obtained^1^. We see from Fig. S3b for 𝜆_IR1_ and Fig. S3d for 𝜆_IR2_ that, near the intensity minima, the *k*_local_ can be more than ten times larger than the allowable highest (*k*_0_ in free space). This confirms that the phase of the super-oscillatory region could locally oscillate much faster than the highest Fourier component. And |*k*_local_|> *k*_0_ is a clear direct indictor of super-oscillations^2^.

Similar super-oscillatory phenomena are also confirmed in the same way for the cases of dielectric SOL, visible SOL and RGB SOL.


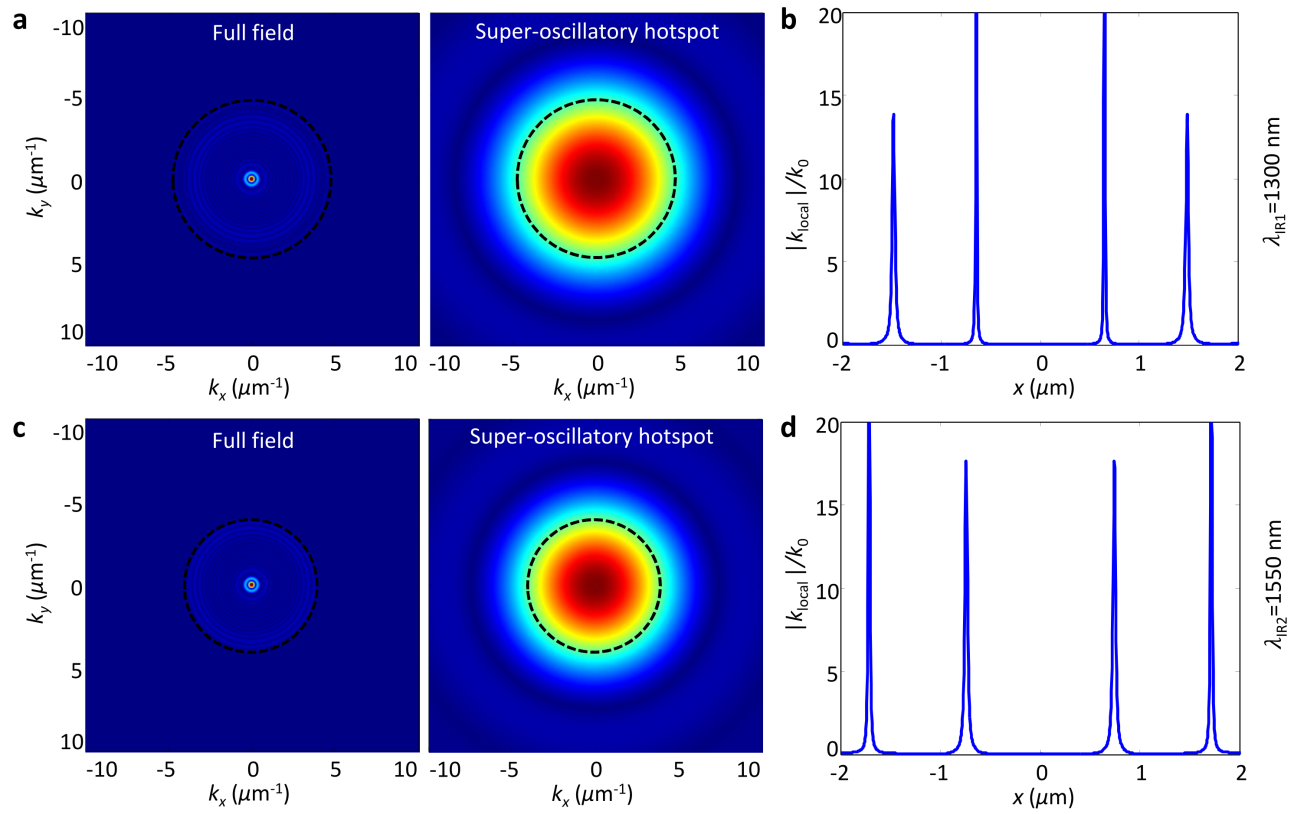


**Figure S3.** (**a, c**) Magnitude of the Fourier spectra $\tilde{E}\left( k_{x},k_{y} \right)$ of the overall electric field in the focal plane of *z*=8 *µ*m (left) and of the super-oscillatory hotspot only (right). (**b, d**) Normalized local wavevectors (|*k*_local_|/*k*_0_) of the focal fields. Due to axial symmetry, only the data on the *x* axis are plotted. Top row for 𝜆_IR1_=1300 nm and bottom row for 𝜆_IR2_=1550 nm.

1. **Optical refractive index of silicon wafer**

The optical properties of the silicon wafer (University Wafer Inc., DSP, 100, p-type) are measured by ellipsometer (J.A. Woollam Co., Inc. WVASE). The fitted complex refractive index is shown in Fig. S4, where the real part at wavelengths of 1300 nm and 1550 nm is n_1300_=3.509 and n_1550_=3.481, while the imaginary part (related to absorption) is extremely small and can be neglected.


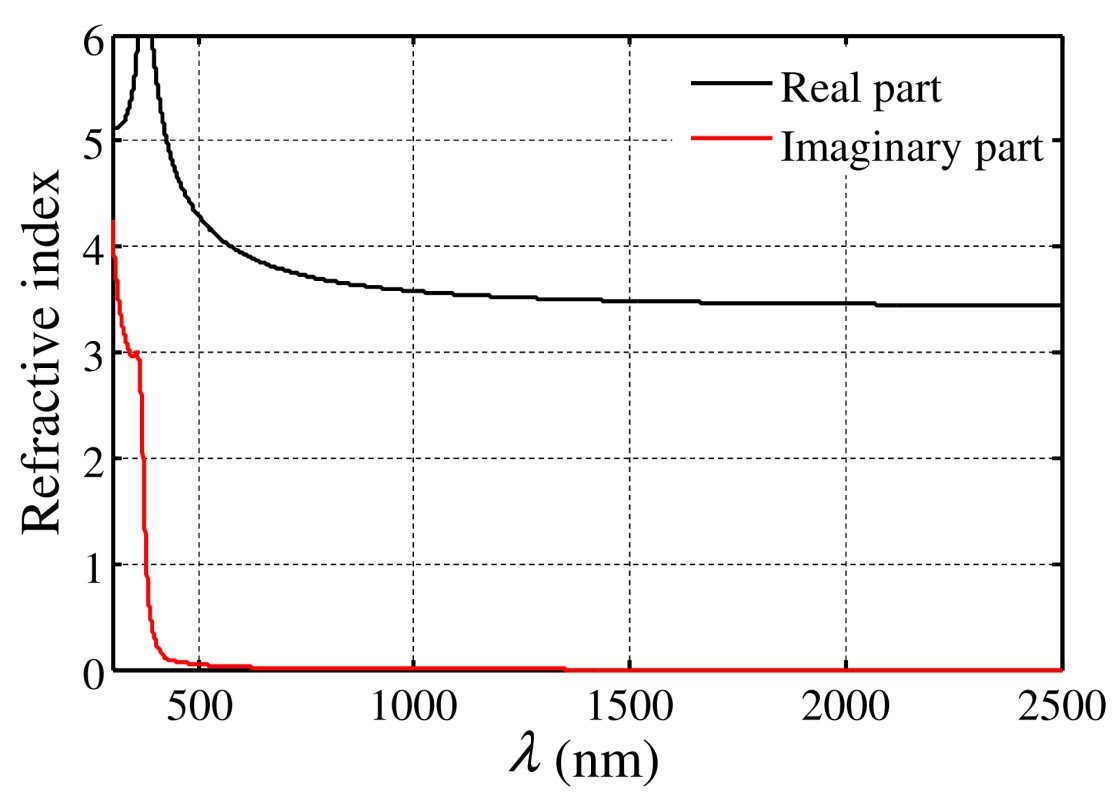


**Figure S4.** Experimentally measured refractive index of silicon wafer.

1. **Comparison between achromatic SOL and binary Fresnel zone plates**

Compared with our achromatic SOL, a conventional diffractive optical element like a binary Fresnel zone plate is highly dispersive and will focus different wavelengths into different axial positions. We give examples in Fig. S5. The radii of alternating transparent and opaque zones are given by the standard formula:

$r_{n}=\sqrt{n\lambda f+\frac{n^{2}\lambda^{2}}{4}}, n=1,2,3,\ldots\ldots$ (2)

where $\lambda$ and $f$ are the working wavelength and focal length respectively.

For an FZP working at 𝜆_1_=690 nm and *f*=18 𝜇m, we used 75 zones in the design and the SEM image is shown in Fig. S5a where the fabrication is conducted on a 100-nm-thick gold film on a glass substrate. Its focusing performance at 𝜆_1_=690 nm and 𝜆_2_=870 nm is characterized by both angular spectrum simulation and experiment which agree well with each other, as shown in Fig. S5b. Indeed, the focal spot for 𝜆_1_ is located at *z*=18 𝜇m as predicted, but for 𝜆_2_ hotspot shifts much closer to the FZP and no hotspot can be observed at *z*=18 𝜇m. Similarly, for the fabricated 59-zone FZP working for 𝜆_2_=870 nm and *f*=18 𝜇m as shown in Fig. S5c, the focal spot is formed at *z*=18 𝜇m for 𝜆_2_, but shifts to be farther away from the FZP for shorter wavelength 𝜆_1_, as seen in Fig. S5d. The theoretical energy concentration ratio at *z*=18 𝜇m is 13.8% and 13.6% respectively, for the two design wavelengths, which is much higher than that of achromatic SOL with similar structural parameters in terms of mask size and focal length, as discussed in the main text. When not used at the design wavelength, the energy concentration ratio for the two FZPs is 0.4% and 0.14%. While the efficiency of the SOL is worse than an FZP working at its design wavelength (an inevitable consequence of super-oscillatory focusing), it has noticeably better efficiency at the second wavelength of interest.


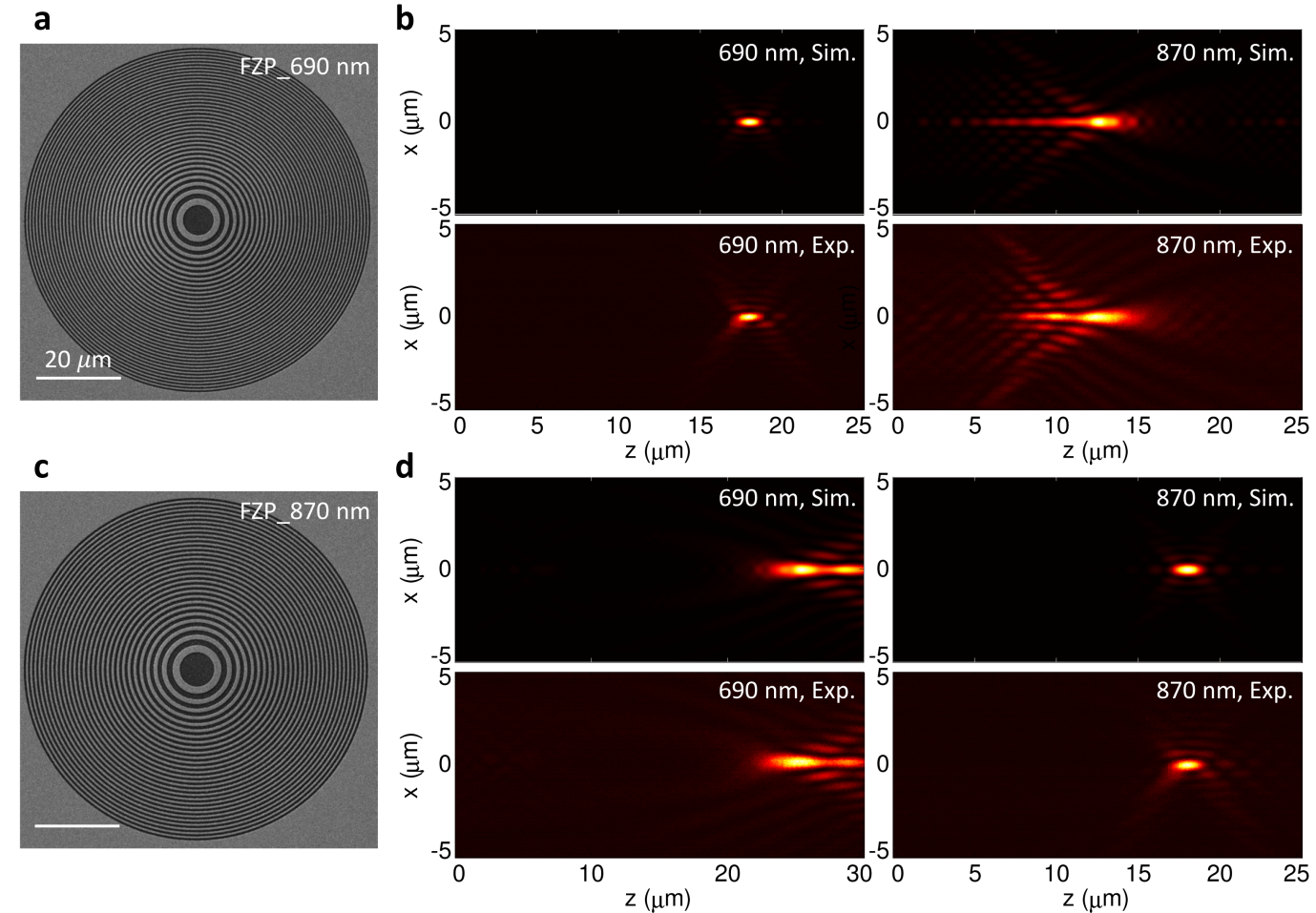


**Figure S5.** (**a**) SEM image of the FZP designed for 𝜆_1_=690 nm and focal length of 18 𝜇m. (**b**) Simulation calculated (top) and experimentally measured (bottom) diffraction patterns in the longitudinal cross-sections for 𝜆_1_=690 nm and 𝜆_2_=870 nm. (**c**) SEM image of the FZP with same focal length of 18 𝜇m for 𝜆_2_=870 nm. (**d**) Simulation calculated (top) and experimentally measured (bottom) diffraction patterns in the longitudinal cross-sections for 𝜆_1_=690 nm and 𝜆_2_=870 nm.

1. **Evolution of diffraction patterns at intermediate wavelengths**


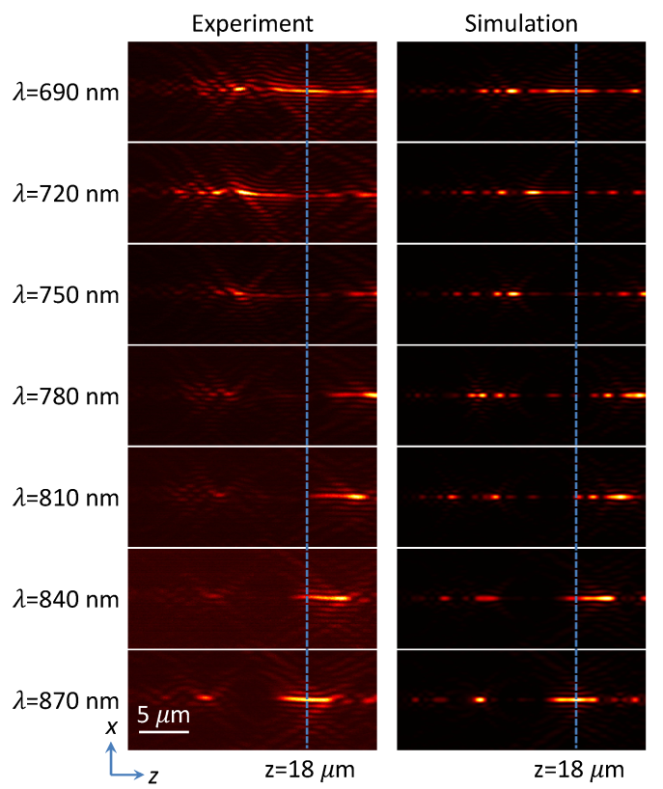


**Figure S6.** Longitudinal cross-section diffraction patterns at tunable wavelengths from 690 nm to 870 nm with a step of 30 nm. (Left column) Experimental data. (Right column) Simulation data obtained from angular spectrum calculations. Good agreement is found between overall profiles and evolution dynamics of the hotspots. The vertical dashed blue lines indicate the designed achromatic focal plane.

1. **Superachromatic SOL design for four wavelengths**


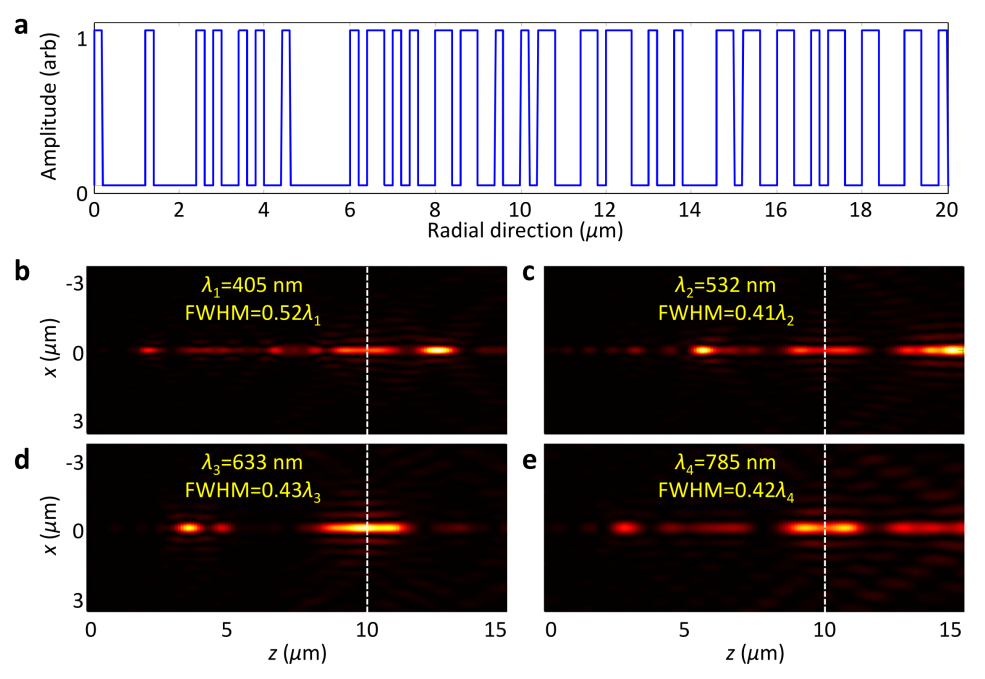


**Figure S7.** (**a**) Binary transmittance of the superachromatic SOL working for four wavelengths: *λ*_1_=405 nm, *λ*_2_=532 nm, *λ*_3_=633 nm and *λ*_4_=785 nm. (**b-e**) Simulated diffraction patterns in the longitudinal plane for *λ*_1_ (**b**), *λ*_2_ (**c**), *λ*_3_ (**d**) and *λ*_4_ (**e**). The FWHM of the super-oscillatory hotspot at designed working distance of *z*=10 *µ*m is also given.

**References**

1. Berry MV, Dennis MR. Natural superoscillations in monochromatic waves in D dimensions. *J Phys A Math Theor* 2009; **42**, 022003.
2. Dennis MR, Hamilton AC, Courtial J. Superoscillation in speckle patterns. *Opt Lett* 2008; **33**, 2976–2978.
